# Sex differences in the neurobiology of fear conditioning and extinction: a preliminary fMRI study of shared sex differences with stress-arousal circuitry

**DOI:** 10.1186/2045-5380-2-7

**Published:** 2012-04-16

**Authors:** Kelimer Lebron-Milad, Brandon Abbs, Mohammed R Milad, Clas Linnman, Ansgar Rougemount-Bücking, Mohammed A Zeidan, Daphne J Holt, Jill M Goldstein

**Affiliations:** 1 Department of Psychiatry, Harvard Medical School & Massachusetts General Hospital, 149 13th St, Charlestown, MA, 02129, USA; 2 Departments of Psychiatry and Medicine, Harvard Medical School, Connors Center for Women's Health and Gender Biology, Brigham and Women's Hospital, 75 Francis St., Boston, MA, 02120, USA; 3 P.A.I.N. Group, Department of Anesthesia, Children´s Hospital, Boston, MA, USA; 4 Department of Psychiatry, Centre Hospitalier Universitaire Vaudois and University of Lausanne, 7 rue Saint-Martin, 1003, Lausanne, Switzerland

**Keywords:** Sex differences, Fear extinction, Fear conditioning, fMRI, Stress response circuitry

## Abstract

**Background:**

The amygdala, hippocampus, medial prefrontal cortex (mPFC) and brain-stem subregions are implicated in fear conditioning and extinction, and are brain regions known to be sexually dimorphic. We used functional magnetic resonance imaging (fMRI) to investigate sex differences in brain activity in these regions during fear conditioning and extinction.

**Methods:**

Subjects were 12 healthy men comparable to 12 healthy women who underwent a 2-day experiment in a 3 T MR scanner. Fear conditioning and extinction learning occurred on day 1 and extinction recall occurred on day 2. The conditioned stimuli were visual cues and the unconditioned stimulus was a mild electric shock. Skin conductance responses (SCR) were recorded throughout the experiment as an index of the conditioned response. fMRI data (blood-oxygen-level-dependent [BOLD] signal changes) were analyzed using SPM8.

**Results:**

Findings showed no significant sex differences in SCR during any experimental phases. However, during fear conditioning, there were significantly greater BOLD-signal changes in the right amygdala, right rostral anterior cingulate (rACC) and dorsal anterior cingulate cortex (dACC) in women compared with men. In contrast, men showed significantly greater signal changes in bilateral rACC during extinction recall.

**Conclusions:**

These results indicate sex differences in brain activation within the fear circuitry of healthy subjects despite similar peripheral autonomic responses. Furthermore, we found that regions where sex differences were previously reported in response to stress, also exhibited sex differences during fear conditioning and extinction.

## Background

A substantial literature implicates the amygdala, hippocampus, hypothalamus, medial prefrontal cortex (mPFC) and brain-stem nuclei in the generation of fear responses and in the inhibition and extinction of fear. Recent work has suggested that these regions are dysregulated in anxiety disorders [1-4]. Interestingly, these regions have also been shown to be sexually dimorphic [5-7] and to activate differentially in healthy men and women under stress [4] and during learning paradigms [8-10]. Therefore, understanding sex differences could provide some insight into the differences between men and women in the incidence of anxiety disorders.

Sex differences in the fear circuitry have been reported in both animal studies [11-15] and human studies [16-18] using fear conditioning paradigms. However, these results are inconsistent. Although some studies have reported no sex differences [19,20], others have reported that in humans and rodents, males tend to exhibit higher conditioning responses relative to females [21,22]. As for fear extinction, we recently reported data showing that estradiol significantly enhances extinction recall in female rats and in women [23]. We have also previously reported sex differences during fear conditioning and fear extinction in humans [17] and in rodents [24]. The modulation of arousal by estradiol is consistent with Goldstein and colleagues’ finding of sex differences in the stress response circuitry of the healthy brain [4], which shares brain regions with fear circuitry. However, the neurobiological mechanisms underlying sex differences shared in fear and stress response circuitries have not been previously reported.

Sex differences in the function of the healthy adult brain during a visual stress challenge in a functional magnetic resonance imaging (fMRI) environment has been studied by Goldstein and colleagues. These authors reported that men, compared with women in the late follicular menstrual phase, showed greater blood-oxygenation-level-dependent [25] signal changes in the amygdala, anterior cingulate cortex (ACC), orbitofrontal cortex (OFC), medial prefrontal cortex (mPFC), hippocampus, anterior hypothalamus and periaqueductal gray [4]. These findings were distinct from comparisons of the same women imaged during the early follicular phase [26], suggesting that circulating sex-steroid hormones partially accounted for sex differences in brain activity in these regions [4], which is consistent with other fMRI studies using arousing stimuli [27]. Goldstein’s reported BOLD-signal differences [4] were in the same ventral mPFC (vmPFC) region as our previous study of the fear circuitry [28], suggesting anatomical overlap between sex differences in arousal due to stress and fear.

In the present study, we used fMRI and a fear conditioning and extinction paradigm to investigate sex differences in the fear circuitry of healthy subjects, extending regions of interest [29] to include those that previous work has identified as part of the stress-response circuitry [4]. Our rationale is based on the idea that arousal is a component of fear and stress, suggesting that they share brain circuitry that is highly sexually dimorphic. Thus, we predict similar sex differences in this circuitry whether arousal is caused by fear or stress-related stimuli. The approach of investigating shared brain circuitry across behavioral domains and psychiatric illnesses is in line with the recent NIMH strategic plan associated with the development of the Research Domain Criteria [30,31].

Subjects participated in a previously established 2-day fear conditioning and extinction paradigm [32]. Conditioning and extinction took place on day 1, and extinction recall took place on day 2. Skin conductance response (SCR) was measured as an autonomic index of fear responses, and all testing took place in a 3T fMRI scanner. Based on previous studies, we hypothesized significant sex differences in brain activity in fear responses in the amygdala, vmPFC, and hippocampus during fear conditioning, extinction learning, and extinction recall. We predicted that healthy men would exhibit greater activity in these arousal-mediating regions than women. More specifically, we predicted that men would exhibit greater activation in the vmPFC and hippocampus and less activation in the amygdala and dACC during extinction recall. Regarding fear conditioning, we predicted greater amygdala and dACC activations in women compared with men and greater vmPFC activation in men compared with women.

## Methods

### Subjects

The sample consisted of 12 healthy women and 12 healthy men who were recruited from the local community via advertisements for two previously published neuroimaging studies [28,33] and reanalyzed to test our hypotheses. One of the original studies was not initially designed to investigate sex differences in fear extinction, therefore women were in different phases of the menstrual cycle (4 follicular phase, 4 late luteal phase, 4 unknown). Table 1 shows that subjects are right-handed and primarily Caucasian with a relatively high education level (at least some college, on average). Men were older and had more years of education. We controlled for these differences when comparing males and females (see below).

**Table 1:** Demographics information about the study subjects

	**Female (n = 12)**	**Males (n = 12)**	**P=**
**Age**	22.1 (SD: 2.6)	26 (SD: 5.0)	0.02
**Years of Education**	15.4 (SD: 1.4)	16.8 (SD: 1.8)	0.04
**Ethnicity**			
Caucasian	10	11	
Asian	2	0	
Hisp/Black	0	1	
**Behavioral measure of anxiety**			
STAI T	30.6 (SD: 6.1)	33.1 (SD: 1.8)	0.5
STAI S	31.5 (SD: 9.4)	29.8 (SD: 6.2)	0.6
**Phases of menstrual cycle**			
Luteal	4	N/A	
Follicular	4	N/A	
unknown	4		

Subjects were excluded if they had neurologic, endocrinologic, or other medical conditions affecting central nervous system function. Subjects were also screened for Axis-I psychiatric disorders, including substance-use disorders, using the Structured Clinical Interview for DSM-IV [34]. There were no significant sex differences in anxiety measures (see Table 1). No participant was using psychoactive or other potentially confounding drugs or medications, and women had abstained from oral contraceptives or hormone replacement for at least three months. After a complete description of the procedures, written informed consent was obtained from all subjects in accordance with the requirements of the Partners Healthcare Human Research Committee.

### Conditioning and extinction procedure

**Figure 1:**
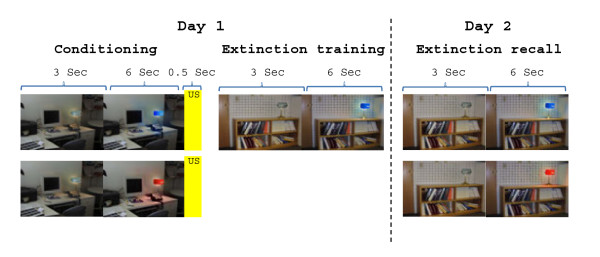
**Illustration of the experimental fear conditioning and extinction protocol used in our experiment.** Adapted from Zeidan et al., 2011. Note that the CS- (represented in a third color of light (yellow) is not shown in this figure for simplicity.

The two-day fear conditioning and extinction procedures have been described elsewhere [35] (see Figure 1). Briefly, two digital photographs of rooms (an office and a library) were the visual contexts in which a lamp was switched from the off position (no color) to one of three colored lights (red, yellow, blue), constituting the conditioned stimuli (CSs). All images were displayed on a computer monitor located approximately two feet behind the subject and viewed on a mirror while the subject was in a 3 T MRI scanner. The unconditioned stimulus (US) was a 500 ms electric shock delivered through electrodes attached to the second and third fingers of the right hand. The subjects had previously selected a shock intensity they found “highly annoying but not painful” [36,37]. The electrodes were attached to the fingers during each phase of the study, but the US was presented only during conditioning. On day 1, subjects underwent the habituation phase in which the conditioning context and the extinction context were displayed four times while each CS was presented two to three times. In the conditioning phase, the (to later be) extinguished conditioned stimulus (CS + E) and the (to later not be extinguished) conditioned stimulus (unextinguished CS + or CS + U) were each presented eight times with 62.5% partial reinforcement (five shocks each), while the conditioned stimulus that was never followed by a shock (CS-) was intermingled and presented 16 times. All CSs were presented in the same context. The selection of CS + and CS- colors was pseudorandom and counterbalanced across subjects. After conditioning, subjects were briefly interviewed to ensure the CS-US pattern was observed. All subjects were aware of the CS-US contingency. The extinction phase immediately followed in which the CS + E and the CS- were each shown 16 times in a new, “safe” context. On day 2, the recall phase was in the extinction context and included the presentation of the CS + U along with the CS + E and CS-.

### Psychophysiological measures

During each trial, the context images were presented for nine seconds: three seconds with the light off immediately followed by six seconds in combination with the CS. The mean inter-trial interval was 15 seconds. Skin conductance responses (SCR) were calculated by subtracting the maximum response during cue presentation from the average response of the two seconds immediately preceding context onset. The SCR values were then square-root transformed to reduce heteroscedasticity. Skin conductance levels [38] were measured during the five seconds preceding the onset of each habituation session trial and then averaged across eight trials to yield a baseline SCL.

To evaluate the amount of fear during the different phases of the experiment, SCR to the CS + and SCR to the CS- were compared as follows: during conditioning, SCR to the CS + was compared to CS-; during extinction, SCR to the CS + E was compared to CS-; and during extinction recall, SCR to the CS + E was compared to CS + U. All data are reported as means ± the standard error of the mean (S.E.). A repeated-measures analysis of variance [39] was used to analyze data across experimental phases. A Student’s *t*-test was used when appropriate*.*

### Image acquisition

Image acquisition parameters were identical to previous reports [28,40,41]. A Trio 3.0-Tesla whole-body, high-speed imaging device with a 12-channel gradient head coil was used (Siemens Medical Systems, Iselin, New Jersey). An automated scout image was obtained and shimming procedures were performed followed by high-resolution, three-dimensional magnetization prepared rapid gradient echo sequences (repetition time [TR]/echo time [TE]/flip angle = 7.25 ms/3 ms/7^o^; 1 mm X 1 mm in plane X 1.3 mm), which were collected for spatial normalization and positioning the subsequent scans. Registration of individual functional scans was based on T1 (TR/TE/flip angle = 8 sec/39 msec/90^o^) and T2 (TR/TE/flip angle = 10 sec/48 msec/120^o^) sequences. fMRI images were acquired with gradient–echo T2*-weighted sequences (TR/TE/flip angle = 3 sec/30 msec/90^o^). The T1, T2, and gradient-echo functional images were all collected in the same plane (45 coronal oblique slices parallel to the anterior-posterior commissure line, tilted 30^o^ anterior) with the same slice thickness (3 mm X 3 mm X 3 mm) except for the T1 (1 mm X 1 mm X 1 mm).

### Functional MRI data analysis

Each subject’s functional time series was first examined for global-signal artifacts (e.g., artifact caused by head movement) using the Artifact Detection Tool (ART) software package (http://web.mit.edu/swg/art/art.pdf) in order to control for this artifact during first-level statistical analyses. “Outlier” volumes were flagged if the average global-signal intensity of the image (i.e., average signal intensity across all voxels) was more than 3.0 standard deviations from the overall mean for all images (ART z-threshold = 3.0), the scan-to-scan translation movement was more than 0.6 mm or the scan-to-scan rotation movement was more than 0.004 radians. Once flagged, outlier volumes were modeled as regressors of no interest in the first-level general linear model (GLM) following standard fMRI pre-processing procedures using SPM8 software (http://www.fil.ion.ucl.ac.uk/spm/software/spm8).

For this pre-processing procedure, images from each functional run were first slice-timing corrected and realigned to the first image of the run. This procedure generated realignment parameters for each run that were also used as covariates of no interest in the first-level GLM, as well as a mean image for each run. These mean images and the MPRAGE were then co-registered to the mean image of the first imaging run to facilitate later transformation of the series into MNI space. Next, the MPRAGE was segmented and spatially normalized to the T1 MNI305 template included in SPM8 (Montreal Neurological Institute, MNI). The resulting spatial-transformation parameters were applied to the EPI time series to transform them to the common anatomical coordinate space (MNI305), and voxels were re-sliced to a dimension of 2 mm isotropic. Finally, to mitigate the effects of residual spatial-transformation noise, the normalized functional images were smoothed using an 8 mm full-width-at-half-maximum Gaussian kernel.

For the first level GLM, we used an epoch model and modeled context and CS as three-second and six-second events, respectively. During conditioning, we modeled the US as a 0.5-second event. Experimental regressors were convolved with the SPM canonical hemodynamic response function (HRF), but the regressors of no interest (i.e. outliers and motion parameters) were not convolved. The time series was subjected to a 128-second high-pass filter to correct for low-frequency signal drift.

First-level statistical parametric maps were calculated using the general linear model for the contrast of interest across the time window [23]. The Stimulus Factor contrasted all 16 CS + trials vs. all 16 CS- trials in the conditioning phase (CS+ > CS-), the last 4 CS + E trials vs. the last 4 CS- trials in the extinction learning phase (CS + E > CS-), and the first 4 CS + E versus the first 4 CS + U trials in the extinction recall phase (CS + E > CS + U). The first four trials were used for extinction recall because we wanted to minimize any confound introduced by additional extinction learning during this phase, and electrophysiological data from rodents indicates that the vmPFC only signals extinction recall during the beginning of extinction recall [42].

First-level SPM contrasts were then grouped during second-level independent-groups t-tests that compared men and women using age and years of education as covariates. For this analysis, we used an uncorrected voxel-level statistical threshold of p < 0.005, and we report only peak-voxels from clusters of activation within our primary anatomical regions of interest (ROIs; vmPFC, insula, dACC, amygdala, and hippocampus). This statistical threshold was used given our focus on specific anatomical ROIs based on prior studies’ findings and *a priori* hypotheses about where sex differences in brain activation should be found. Following this analysis focusing on previously identified fear conditioning circuitry regions, we conducted a stringent functional-ROI analysis of sex differences in stress-response circuitry regions during fear conditioning.

### Functional ROI construction

Given our hypothesis that the brain’s stress-response circuitry and fear conditioning circuitry partly overlap, sex differences found in previous fMRI studies of the stress-response circuitry should also be apparent during fear conditioning. We identified *a priori* anatomical ROIs using Goldstein and colleagues findings [4,26] of functional sex differences in the following regions during an emotional arousal task that activates the stress-response circuitry: orbitofrontal cortex (OFC), anterior cingulate cortex (ACC), the periaqueductal gray brain stem area [43], hippocampus (HIPP), anterior hypothalamus (HYP), and amygdala (AMG). However, shared functional sex differences within a broad anatomical area (particularly OFC and ACC) do not necessarily suggest any similar function for these regions in both paradigms. Therefore, we further limited our search within these anatomical regions to functional ROIs (spheres) around Goldstein’s (2010) exact coordinates (Table 2). To do this, we created either 8 mm (cortical rois) or 4 mm (subcortical ROIs) spheres using the WFU Pickatlas tool for SPM8. We then examined sex differences in these spheres with an initial uncorrected voxel-level threshold of p < .05, which was corrected to a FWE p-value of p < .05 using small-volume correction. To characterize activation magnitude differences in both anatomical and functional ROIs, we calculated the average GLM beta value for each ROI by averaging across the beta value for all voxels within an ROI.

**Table 2:** Coordinates from Goldstein et al. (2010) used in the anatomical ROI analysis

Region	**X**	**Y**	**Z**
**Dorsal ACG**			
R. BA 32	12	34	28
R. BA 32	14	16	26
R. BA 24	4	18	20
L. BA 32	−2	18	20
L. BA 32	−2	22	28
**Medial PFC**			
R. BA 10	20	66	10
R. BA 10	34	58	−4
L. BA 10	−4	64	20
**Ventral mPFC**			
R. BA 10	4	54	−8
L. BA 10	−4	42	−20
**OFC - BA 1**			
	2	40	−22
**Amygdala**			
Right	18	−2	−14
Left	−18	−4	−14
**Hippocampus**			
Right	30	−24	−8
Left*	−30	−24	−8
**Hypothalamus**			
Right*	4	2	−6
Left	−4	2	−26
**Periaqueductal Gray**			
	0	−30	−2

*Goldstein et al. (2010) did not report significant bi-lateral activation in these regions. Given the role of laterality in fear conditioning in these regions, we created ROls in the contralateral hemisphere of Goldstein and colleague’s significant findings

## Results

### Psychophysiology during conditioning, extinction, and extinction recall

**Figure 2:**
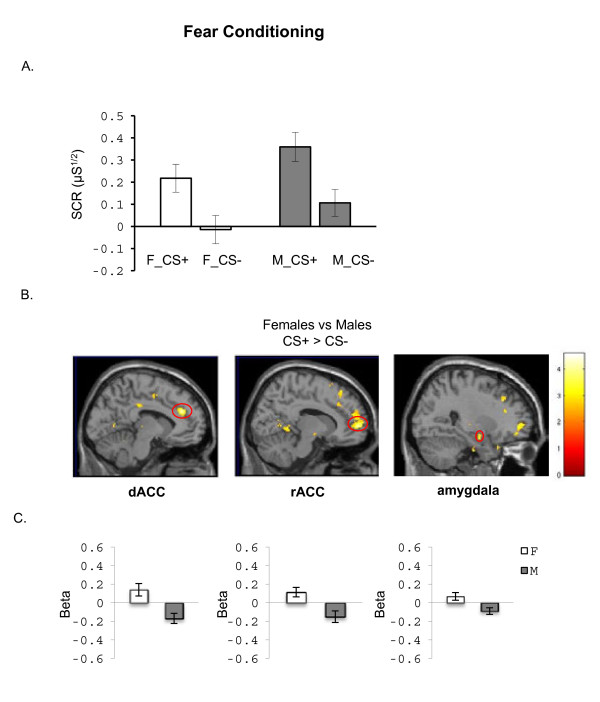
**Differences between men and women in psychophysiological and BOLD measures during fear acquisition. A.** Skin conductance responses (SCR) averaged across the first 4 conditioning trials for the conditioned stimulus that was reinforced, i.e. paired with the shock (CS+) and for the conditioned stimulus not paired with the shock (CS-). **B.** BOLD activation to the CS + vs. CS- contrasting Females vs. Males during fear conditioning is shown. **C.** Mean beta weights extracted from the dorsal anterior cingulate cortex (dACC), rostral anterior cingulate (rACC), and amygdala are shown, to illustrate the direction of activation within group. The threshold display for the maps in B is p < 0.01, uncorrected. M = males; F = females.

Men and women showed differential conditioning. An ANOVA conducted on the SCR data revealed a significant Stimulus main effect (F_(1,22)_ = 30.2, p < 0.001) with greater responses to the CS + than to CS- (first 4 trials of each type during conditioning) in women and men, indicating that both were able to learn the CS-US association. No significant main effects of Group (F_(1,22)_ = 2.9, p = 0.11) or Group X Stimulus interaction (F_(1,22)_ = 0.57, p = 0.81) were observed, indicating no significant sex differences in SCR during conditioning, even though males showed higher SCR than females but not significantly so (see group effect above, at p = .11) (see Figure 2A).

**Figure 3:**
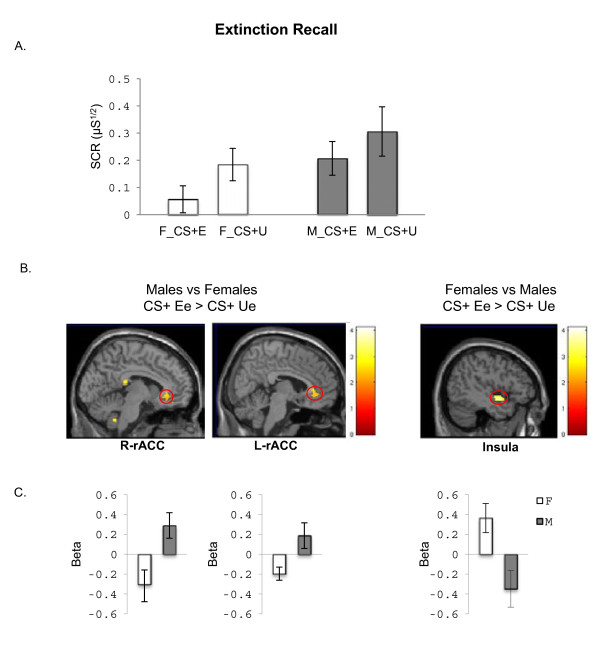
**Differences between men and women in psychophysiological and BOLD measures during extinction memory recall. A.** Skin conductance responses (SCR) averaged across the first 4 extinction recall trials for the extinguished stimulus (CS + E) compared to the unextinguished stimulus (CS + U). **B.** BOLD activation to the CS + E vs. CS + U contrasting Females vs. Males during extinction recall are shown. **C.** Mean beta weights extracted from the left and right rostral ACC and insula are shown, to illustrate the direction of activation within group. The threshold for the maps in B is p < 0.01, uncorrected. M = males; F = females.

During extinction training on day 1, an ANOVA for the late extinction SCR data (last 4 CS + E vs. last 4 CS- trials) revealed no significant main effect of Stimulus (F_(1,21)_ = 0.21, p = 0.65) or Group (F_(1,21)_ = 2.23, p = 0.09) and no significant Group X Stimulus interaction (F_(1,21)_ = 0.14, p = 0.71), suggesting that comparable extinction learning had been achieved in both groups (data not shown). An ANOVA for the early extinction recall SCR data (first 4 CS + E vs. first 4 CS + U trials) also revealed no significant main effects of Stimulus (F_(1,22)_ = 3.19, p = 0.09), Group (F_(1,22)_ = 2.33, p = 0.14) or Group X Stimulus Interaction (F_(1,22)_ = 0.37, p = 0.55) (Figure 3A). Analyses of the extinction retention index, which controls for the level of fear acquired during the conditioning phase [23], revealed no significant group differences in extinction retention confirming that no significant differences between groups during extinction recall (data not shown). Collectively, we did not observe any statistically significant differences between men and women in our psychophysiological measures during any experimental phase. It is important to note that several statistical sex difference trends were observed with men tending to show higher SCR to the CSs throughout conditioning and extinction even though the differences were not statistically significant.

### Blood-oxygen-level dependent (BOLD) responses in anatomical ROIs

During fear conditioning, females responded to the conditioned stimulus with significantly greater activation, relative to males, in the following *a priori* anatomical regions of interest: the dACC, rACC, and the amygdala (see Table 3 for coordinates and statistical results and figure 2B). The average beta values for these ROIs (extracted from the between-group activation maps) show that these sex differences were present in dACC, mPFC and amygdala activation in females and deactivation in males (see Figure 2C).

**Table 3:** BOLD responses from both our anatomical ROI analyses and our functional ROI analyses comparing males and women during the different phases of the study

**Bold responses from anatomical ROI analyses**					
**Phase**	**condition**	**regions**	**coordinates**	**z**	**P(≤)**
conditioning	F > M	L-dACC	−8,38,26	3.54	0.001
		R-rACC	12,58,4	3.44	0.001
		Amyg	28,-6,-16	3.08	0.001
	M > F	non			
Ext learning	F > M	non			
	M > F	non			
Ext Recall	F > M	L-insula	−46,-2,-10	3.15	0.002
	M > F	L-rACC	−6,32,-4	2.79	0.005
**BOLD responses from functional ROI analyses**					
Conditioning	F > M	non			
	M > F	non			
Ext Learning	F > M	dACC	8,12,18	3.62	0.001
		dACC	10,14,20	3.27	0.001
		mPFC	28,54,-4	3.33	0.001
		L-hypotha	−8,2,-6	2.25	0.024
	M < F	hypothalamus	6,4,-6	2.44	0.015
Ext Recall	F > M	non			
	M > F	L-rACC	−6,42,-16	3.16	0.001

ROIs are based on sexually dimorphic areas of the stress response circuitry identified by Goldstein and colleagues (2010).

We did not observe any significant sex differences in our anatomical ROIs during the extinction learning phase. During extinction recall, greater activation in the rostral region of the left rostral ACC (rACC) was observed in males relative to females. A trend in the same direction was also observed in the right rACC, which corresponds to a similar trend in right vmPFC [(6, 34, 0); z = 2.76, p = 0.006]. Additionally, greater insula activation was observed in females relative to males (see Figure 3B, Table 3). The average beta values from rACC showed that males were activating the rACC while females were deactivating this region. In contrast, females were activating insula, while males were deactivating this region (see Figure 3C).

### BOLD responses from functional ROI analysis

We extended our ROI analyses based on the anatomy of the fear-conditioning circuitry by conducting an analysis based on previously reported [4] functional sex differences in the stress-response circuitry, such as the anterior hypothalamus. These data are summarized in Table 3. Although we found sex differences in the fear conditioning circuitry in our anatomical ROIs, no significant sex differences were observed in functional stress-response ROIs (data not shown). Conversely, Table 3 shows that during extinction learning (when no sex differences in fear circuitry were found), males showed significantly greater activation in right hypothalamus, and females showed greater activation in left hypothalamus, dACC, and mPFC. Lastly, extinction recall showed sex differences in fear and stress-response circuitry, as males exhibited significantly higher signal changes relative to females in our functional ROIs (Table 3).

## Discussion

In this study, we used fMRI to investigate sex differences in BOLD-signal changes of healthy subjects exposed to a fear conditioning and fear extinction paradigm. No statistically significant sex differences in SCRs were observed during any experimental phase. However, we note that men exhibited a trend towards generally elevated SCRs during acquisition of conditioned fear responses and the extinction recall test. Regarding the BOLD responses, we observed significant sex differences in several brain regions during fear conditioning and extinction recall. Specifically, during fear conditioning, women showed greater activation in dACC, rACC and amygdala relative to men. During extinction recall, women showed greater activation in the insula cortex relative to men, while men showed greater activation in the rACC region of the mPFC relative to women. Moreover, we found that regions where sex differences were previously identified in response to stress [4] also exhibited sex differences during fear conditioning and extinction, including the anterior hypothalamus.

Sex differences at the behavioral level have been reported in humans and rodents across a number of paradigms such as fear conditioning, active avoidance, conditioned taste aversion and eye blink conditioning [14]. Some studies report increased conditioned responding in males rats during fear learning [21], which is consistent with our findings of sex differences in SCR, whereas others found no significant sex differences in fear acquisition [20]. We previously reported that men show elevated conditioned fear responses relative to women [17,23]. While not statistically significant in this study, men exhibited a trend towards elevated conditioned fear responses. The lack of a statistically significant different sex difference may be due to variability of endocrine status or use of contraceptives among the women in our sample. That is, we recently demonstrated that estradiol significantly enhances fear extinction recall and its neural correlates during fear extinction [33], and others have reported that contraceptives can impact learning and memory [44]. Moreover, it has been shown that estradiol facilitated contextual fear extinction via estradiol’s effect on hippocampal long-term potentiation in rats [45]. One important caveat is that in our data, men also showed a trend towards increased skin conductance responses to the CS- compared with the women. This would suggest enhanced general skin conductance reactivity in men relative to women that may not be specific to fear learning or extinction per se given that they are higher across conditions. Future studies will need to further investigate the role of sex-steroid hormones in understanding variability due to gender during conditioning and the neural responses of the fear extinction network.

Using an anatomical ROI analysis approach, we observed sex differences in fear circuitry activation. Women exhibited greater amygdala, mPFC and dACC activation during fear acquisition and greater insula activation during extinction recall. In contrast, men showed significantly greater activation within the rACC, which is in close proximity to the locus we previously reported showing increased activation during fear extinction [35,46,47]. The mPFC and dACC have been implicated in pain processing, conflict monitoring and error processing, fear expression, and appraisal of emotionally salient stimuli [41,48-50]. The amygdala is also well known for signaling novelty and mediating emotional learning such as fear conditioning [51]. The vmPFC has been implicated in emotion regulation and fear extinction recall [2,52]. The increased mPFC activation in men during extinction recall predicted facilitated fear extinction recall in men and fear responses in women, and the increased amygdala and dACC activation in women during fear conditioning again predicted facilitated fear responses in women during this phase. The behavioral data showed lack of sex differences in fear responses. Thus brain activity *differences* in neural responses may be contributing to producing *similar* behavioral responses suggesting that men and women use different neural strategies to produce homeostasis in the brain in response to fear. This was similar to Goldstein’s previous findings of sex differences in neural responses to stress to maintain homeostasis in the brain in response to stress, which was dependent on menstrual cycle phase in the women [4]. Further studies are needed to explore whether men and women use different neural networks to acquire and control fear to a similar degree.

In fact, the functional ROI analyses revealed overlap between sex differences in stress-arousal circuitry activation and sex differences in fear-arousal circuitry reported here. For example, Goldstein and colleagues [4] reported that men, compared with women in the late follicular menstrual cycle phase, exhibited significantly greater BOLD-signal changes in response to negative versus neutral stimuli in ACC, OFC, mPFC, anterior hypothalamus, hippocampus and periaqueductal gray. Although the regions of activation overlap, data from the present study indicated that women exhibited significantly greater BOLD-signal changes, compared to men, during fear conditioning in a number of these brain regions. However, no women in the current study were scanned during the mid-cycle menstrual phase, as distinct from all women in the Goldstein study who contributed to the sex difference effect were scanned during this phase. Men in both studies showed hyperactivation in vmPFC, other orbitofrontal regions, and right hypothalamus. In our study, hyperactivation was observed during extinction recall even without controlling for menstrual cycle phase. The differences between our findings and those reported by Goldstein and colleagues may be due to differences in levels of sex hormones, particularly estradiol and/or progesterone [33], but this hypothesis needs further investigation. Nevertheless, the approach underscores the importance of analyzing sex differences in shared brain circuitry across behavioral domains for understanding psychiatric disorders.

## Conclusions

In summary, we present data showing sex differences in the functional responses of the fear-response circuitry during fear conditioning and extinction recall that overlap in location with previously reported sex differences in the functional response of the stress-response circuitry during arousal. Although sex differences at the neural level (i.e., BOLD-signal change) were robust, differences at the psychophysiological level (i.e., SCR) were less reliable or non-existent. Future studies examining the influence of sex hormones such as estradiol, progesterone and testosterone on sex differences are needed to further advance this line of research.

## List of Abbreviations

mPFC = medial prefrontal cortex; fMRI = functional magnetic resonance imaging; SCR = skin conductance responses; BOLD = blood-oxygenated-level-dependent; SPM8 = Statistical Parametric Mapping 8; ACC = anterior cingulate cortex; rACC = rostral anterior cingulated cortex; OFC = orbitofrontal cortex; vmPFC = ventral medial prefrontal cortex; CS = conditioned stimulus; US = unconditioned stimulus; CS + E = extinguished conditioned stimulus; CS + U = unextinguished stimulus; CS = conditioned stimulus that was never paired with US; SCL = skin conductance levels; ANOVA = repeated-measures analyses of variance; 3 T = 3 tesla; GLM = general linear model; ROIs = regions of interest; PAG = periaqueductal gray; HIPP = hippocampus; HYP = anterior hypothalamus; AMG = amygdala; dACC = dorsal anterior cingulate cortex.

## Competing interest

Dr. Mohammed R Milad received consulting fees from Microtranspondor for a project unrelated to the one described in this manuscript. The remaining authors declare no conflicts of interest.

## Authors’ contributions

All authors have made substantive intellectual contributions to the study. All authors contributed to: 1) the conception and design of the study, or acquisition of data, or analyses and interpretations of data; 2) drafting the manuscript or revising it critically for important intellectual content, and 3) final approval of the version to be publish.
